# Mitochondrial-Derived Vesicles Protect Cardiomyocytes Against Hypoxic Damage

**DOI:** 10.3389/fcell.2020.00214

**Published:** 2020-04-17

**Authors:** Binghu Li, Hongliang Zhao, Yue Wu, Yu Zhu, Jie Zhang, Guangming Yang, Qingguang Yan, Junxia Li, Tao Li, Liangming Liu

**Affiliations:** State Key Laboratory of Trauma, Burns and Combined Injury, Department 2, Research Institute of Surgery, Daping Hospital, Army Medical University, Chongqing, China

**Keywords:** mitochondrial-derived vesicles, myocardial ischemia, mitochondrial, hypoxia, apoptosis

## Abstract

Myocardial ischemia is a condition with insufficient oxygen supporting the heart tissues, which may result from myocardial infarction or trauma-induced hemorrhagic shock. In order to develop better preventive and therapeutic strategies for myocardial ischemic damage, it is important that we understand the mechanisms underlying this type of injury. Mitochondrial-derived vesicles (MDVs) have been proposed as a novel player in maintaining mitochondrial quality control. This study aimed to investigate the role and possible mechanisms of MDVs in ischemia/hypoxia-induced myocardial apoptosis. H9C2 cardiomyocytes were used for the cellular experiments. A 40% fixed blood volume hemorrhagic shock rat model was used to construct the acute general ischemic models. MDVs were detected using immunofluorescence staining with PDH and TOM20. Exogenous MDVs were reconstituted *in vitro* from isolated mitochondria under different hypoxic conditions. The results demonstrate that MDV production was negatively correlated with cardiomyocyte apoptosis under hypoxic conditions; exogenous MDVs inhibited hypoxia-induced cardiomyocyte apoptosis; and MDV-mediated protection against hypoxia-induced cardiomyocyte apoptosis was accomplished via Bcl-2 interactions in the mitochondrial pathway. This study provides evidence that MDVs protect cardiomyocytes against hypoxic damage by inhibiting mitochondrial apoptosis. Our study used a novel approach that expands our understanding of MDVs and highlights that MDVs may be part of the endogenous response to hypoxia designed to mitigate damage. Strategies that stimulate cardiomyocytes to produce cargo-specific MDVs, including Bcl-2 containing MDVs, could theoretically be helpful in treating ischemic/hypoxic myocardial injury.

## Introduction

Myocardial ischemia is a condition where the heart tissue receives an insufficient amount of oxygen. Myocardial ischemia may result from myocardial infarction or even trauma-induced hemorrhagic shock. Ischemic myocardial injuries seriously affect the prognosis and survival of patients (Angele et al., 2008). However, the underlying mechanisms of myocardial ischemic or hypoxic injury have not been fully elucidated. That said, apoptosis has been identified as one of the major contributing factors to this pathology (Xu et al., 2019). Previous studies have shown that inhibiting the apoptosis signaling pathway exerts a cardioprotective effect in ischemic animal models (Xu et al., 2015; Lin et al., 2016). Despite some progress, the specific signaling pathway used during this type of injury has not been fully elucidated.

It is well established that the hearts possess large numbers of mitochondria (Stanley et al., 2005), and that the maintenance of normal mitochondrial function plays an important protective role in ischemic myocardial injury (Jin et al., 2018; Antonucci et al., 2019). Mitochondrial quality control mechanisms, including the cytosolic ubiquitin-proteasome system and mitophagy, function to maintain the normal physiological function of the cells. In addition, mitochondrial-derived vesicles (MDVs), secreted by the mitochondria, have been identified as a novel player in mitochondrial quality control. These MDVs are thought to function by delivering specific mitochondrial contents to late endosome/multivesicular bodies (Soubannier et al., 2012a; Soto-Heredero et al., 2017). More recently, MDVs have been reported to transport mitochondrial autoantigens to the surface of the cell membrane to trigger the immune response (Matheoud et al., 2016) and deliver superoxide dismutase (Sod2) to bacteria-containing phagosomes to produce hydrogen peroxide and kill invading bacteria (Abuaita et al., 2018). However, the role of MDVs is far from being completely understood. A recent study showed that MDV production in myocardial tissues occurs at low basal levels and is up-regulated, within a few hours, in response to prototypical mitochondrial stressors acting upstream of mitophagy (Cadete et al., 2016). Thus, it is believed that MDVs may play a critical role in the maintenance of the heart during physiological or pathological stress. However, the specific role of MDVs in the heart is still unclear. In this study, we investigated the role and possible mechanisms of MDVs in protecting cardiac tissues from ischemia/hypoxia-induced myocardial apoptosis.

## Materials and Methods

### Reagents

Dulbecco’s modified Eagle’s medium (DMEM) and fetal bovine serum (FBS) were purchased from Hycolon (Logan, UT, United States). PKH26 Red Fluorescent Cell Linker Kits were obtained from Sigma-Aldrich (St. Louis, MO, United States). Calcein-acetomethoxy ester and MitoTracker Green FM were from Molecular Probes (Carlsbad, CA, United States). Caspase 3/9 activity assay kit, Cell Mitochondria Isolation Kit, ATP Assay Kit, one step TUNEL Kit and Cell Count Kit-8 were from Beyotime (Beijing, China). Reactive oxygen species Assay Kit was from Nanjing Jiancheng Bioengineering Institute (Nanjing, China). Antibody against TOM20 (NBP1-81556; 1:200) for immunofluorescence staining was from Novus Biologicals (Littleton, CO., United States). Antibody against Bcl-2 (sc-578; 1:50) for immunofluorescence staining was from Santa Cruz Biotechnology (Santa Cruz, CA, United States). Antibodies against TOM20 (42406; 1:1000), VDAC (4866; 1:1000), cleaved-caspase 3 (9661; 1:1000), PPAR (9542; 1:1000), caspase 9 (9508; 1:1000), and Cytochrome C (11940; 1:1000) were from Cell Signaling Technology (Beverly, MA, United States). Antibodies targeting PDH (ab110333; 1:500), SDHA (ab137040; 1:1000), caspase 12 (ab62484; 1:1000), CHOP (ab11419; 1:1000), and Bcl-2 (ab59348; 1:1000 for WB) were from Abcam (Burlingame, CA, United States). Antibodies targeting GAPDH (MA1-16757; 1:1000) and β-actin (MA1-91399; 1:7000) were from Thermo Fisher (Wilmington, DE, United States). IRDye 800CW conjugated secondary antibodies were from LI-COR (Lincoln, NE, United States). Alexa Fluor 350-/Alexa Fluor 488-/Cy3- labeled secondary antibodies were from Beyotime (Beijing, China).

### Cell Culture and Hypoxia

The cardiomyocyte cell line H9C2 was obtained from the American Type Culture Collection (ATCC, Manassas, VA, United States) and cultured in DMEM supplemented with 10% FBS in a humidified, 5% CO_2_/95% air atmosphere at 37°C. Hypoxic treatments were performed as previously described (Yang et al., 2017). Briefly, H9C2 cells were transferred into a hypoxia culture compartment (MIC-101; Billups-Rothenberg, Del Mar, CA, United States), bubbled with 95% N_2_ and 5% CO_2_ at 10 L/min for 15 min, and then equilibrated for 10 min. This procedure was repeated five times until the O_2_ concentration in the chamber was 0.2%. H9C2 cells were maintained under these conditions as indicated and used for subsequent experiments.

### Animal Management

All animal procedures were performed in accordance with the NIH guidelines and were approved by the Laboratory Animal Welfare and Ethics Committee of the Third Military Medical University. Protocols were all designed according to the guidelines for the ethical use of animals. Sprague-Dawley (SD) rats (200–240 g) were purchased from the Animal Center of the Research Institute of Surgery and starved for 12 h, apart from water which was allowed *ad libitum* before the experiment.

### Isolation of Mitochondria and Reconstitution of MDV *in vitro*

To construct various MDVs from the different hypoxic conditions, mitochondria were isolated from *ex vivo* Langendorff-perfused rat hearts. Briefly, rats were anesthetized using an intraperitoneal injection of pentobarbital sodium (30 mg/kg), and anesthesia was confirmed by needle stimulation without response. The hearts were then harvested and immediately submerged in ice-cold Ca^2+^-free Tyrode Solution (137 mM NaCl, 5.4 mM KCl, 1.2 mM MgCl_2_, 10 mM HEPES, 10 mM glucose, 1.2 mM NaH_2_PO_4_). The aorta was swiftly cannulated with a 21-gage cannula and the heart was retroactively perfused with Tyrode Solution (137 mM NaCl, 5.4 mM KCl, 1.2 mM MgCl_2_, 10 mM HEPES, 10 mM glucose, 1.2 mM NaH_2_PO_4_, and 1.2 mM CaCl_2_) aerated with a mixture of O_2_ (95%) and CO_2_ (5%) in order to maintain O_2_ levels at 800 nmol/mL using a murine Langendorff perfusion apparatus. After a stabilization period of 20 min, the perfusion buffer was switched to the Tyrode Solutions containing various O_2_ concentrations (normoxia: 800 nmol/mL; mild hypoxia: 550 nmol/mL; heavy hypoxia: 300 nmol/mL), which have been proven to induce various myocardial injuries in previous studies (Anttila et al., 2017) for an additional 40 min of perfusion. Then, the hearts were cut into small pieces and homogenized in cold isolation buffer (20 mM HEPES, 220 mM mannitol, 68 mM sucrose, 80 mM KCl, 0.5 mM EGTA, 2 mM magnesium acetate, supplemented with protease inhibitors, pH 7.4) for mitochondrial isolation which was done using a protocol adapted from a previous study (McLelland et al., 2016). After centrifuging at 600 × *g* for 5 min, the post nuclear supernatant was collected and centrifuged again at 1,000 × *g* for 10 min. These supernatants were collected and centrifuged again at 7,000 × *g* for 10 min. The pellet (mitochondria) was then resuspended in a large volume of isolation buffer, centrifuged again, and then stored in isolation buffer on ice for MDV formation. The supernatant was then centrifuged at 200,000 × *g* for 90 min. The resulting supernatant (organelle-free supernatant) was stored on ice for use in MDV formation.

MDVs were reconstituted using the methods from a previous study (Soubannier et al., 2012b). Briefly, a 1 mL-reaction system containing 30 mg mitochondria, 3 mg/mL organelle-free supernatant, and ATP regenerating mixture (1 mM ATP, 5 mM succinate, 80 μM ADP, and 2 mM K_2_HPO_4_, pH 7.4) was incubated at 37°C for 2 h and then diluted in 10 mL PBS and centrifuged at 12,000 × *g* for 10 min at 4°C. Supernatants were filtered through a 0.22−μm filter (Millipore), the filtrates were centrifuged at 110,000 × *g* for 80 min at 4°C and the resultant pellets were comprised of the MDVs.

### Acute General Ischemic Models

To construct the acute general ischemic models, 40% fixed blood volume hemorrhagic shock models were adopted. Rats were anesthetized with intraperitoneal injection with sodium pentobarbital (30 mg/kg body weight) until they did not respond to a needle stimulus. The right femoral artery and vein were catheterized with polyethylene catheters for bleeding and drug administration, respectively. After 10 min of stabilization, rats in the ischemia group underwent a 40% hemorrhage within 40 min (the total estimated blood volume was 70 mL/kg body weight). Rats in the control group underwent identical management without hemorrhage. In the *in vivo* study of the role of MDVs, rats were grouped into three groups: control (*n* = 6), ischemia (after 40% hemorrhage, treated with 200 μL PBS, *n* = 6), and MDV group (after 40% hemorrhage, treated with 200 μL heavy hypoxic MDVs [h-MDVs, 600 μg/kg, which was referred to a previous study (Monsel et al., 2015)], *n* = 6). After 3h-observation, serum was collected for troponin T (TnT), creatine phosphokinase-MB (CK-MB), and lactate dehydrogenase (LDH) analysis on the automatic biochemical analyzer (DX800; Beckman Coulter, Fullerton, CA) housed in the Clinical Laboratory of our Hospital. Hearts were harvested after the animals were sacrificed for TUNEL staining.

### Western Blot

The total proteins of cells were extracted with RIPA lysis solution and mitochondrial protein were obtained by Cell Mitochondria Isolation Kit according to the manufacturer’s instruction. The concentration of protein was determined using a BCA kit. Protein samples were separated by SDS-PAGE, transferred to a NC membrane and blocked with TBS containing 0.05% Tween-20 (TBST) and 5% non-fat milk powder for 2 h. The membranes were then incubated with appropriate primary antibodies overnight at 4°C. After 5 min × 3 washes with TBST, membranes were incubated with secondary antibodies for 2 h at room temperature. After 5 min × 3 washes with TBST, membranes were detected by Odyssey CLx Imaging System. Band density was measured using Image J and normalized to β-actin or VDAC expression, respectively.

### Immunofluorescence

Cells were fixed in 4% paraformaldehyde for 15 min at room temperature, permeabilized using 0.1% Triton-X for 10 min, and then blocked with 1% bovine serum albumin for 20 min. Cells were incubated with primary antibody overnight at 4°C. Secondary antibodies were then added for 1 h at 37°C. Cells were analyzed using a confocal microscope (Leica). For MDV detection, immunofluorescence was performed as previously described (McLelland et al., 2014). Briefly, cells were fixed in 5% paraformaldehyde at 37°C for 15 min and quenched in NH_4_Cl (50 mM) for 10 min. Cells were then permeabilized with 0.3% Triton-X. With the staining and evaluation steps performed as described above, vesicles greater than 300 nm in size and PDH positive or TOM20 positive alone were identified as MDVs (Sugiura et al., 2014).

### Apoptosis Detection

Cell apoptosis was detected by the one step TUNEL kit according to the manufacturer’s instruction. Briefly, H9C2 cells or cardiac tissues were fixed with 4% paraformaldehyde for 30 min at room temperature and permeabilized with 0.15% Triton-X for 5 min on ice followed by TUNEL for 1 h at 37°C. Nuclei were counterstained with DAPI. Images were acquired using a confocal fluorescent microscope. The cells with green fluorescence were defined as apoptotic cells.

### Measurement of Caspase 3/9 Activity

Caspase 3/9 activity assay was performed according to the manufacturer’s instruction. Briefly, after lysed with lysis buffer and centrifugation, 50 μL supernatants were added to a reaction buffer containing 10 μL 2 mM Ac-DEVD-pNA (Ac-LEHD-pNA for caspase 9 activity assay) for 2 h-incubation at 37°C. The absorbance was measured by a spectrometer at 405 nm. The specific caspase 3/9 activity was normalized for total protein and then expressed as fold of the baseline caspase 3/9 activity of control cells.

### Cell Viability Assay

Cell viability was detected by Cell Count Kit-8, which allows sensitive colorimetric assays for the determination of the number of viable cells in cell proliferation and cytotoxicity assays by using WST-8 [2-(2-methoxy-4-nitrophenyl)-3-(4-nitrophenyl)-5-(2,4-disulfophenyl)-2H-tetrazolium, monosodium salt]. Briefly, cells were seeded in 96-well flat-bottomed plates at 5 × 10^3^ cells/well and treated as indicated. And then the original medium was replaced by 100 μL 10% FBS DMEM medium contains 10 μL CCK-8 and incubated at 37°C for 2 h. The absorbance was determined using a microplate reader at 450 nm.

### ATP Detection

The level of ATP was measured using ATP Assay Kit according to the manufacturer’s instructions. Briefly, harvested cultured cells were lysed with a lysis buffer, followed by centrifugation at 12,000 × *g* for 5 min at 4°C. The supernatant was collected for protein concentration detection and ATP detection. Protein concentration was measured by BCA method. The level of ATP was determined by mixing 20 μL of the supernatant with 100 μL of ATP detection working buffer. The emitted light measured using a multi-detection microplate reader. Level of ATP was normalized for total protein.

### Intracellular Reactive Oxygen Species Assay

Assay of intracellular reactive oxygen species was using 2′,7′-dichlorofluorescein diacetate. Briefly, cells were cultured on confocal dish at 2 × 10^4^ cells/dish. After treated as indicated, cells were incubated with 10 μM DCFH-DA for 20 min at 37°C. After three washes with PBS, cells were observed using a fluorescence microscope.

### Measurement of Mitochondrial Permeability Transition Pore Opening

To analyze the opening of mitochondrial permeability transition pore (mPTP), cells were cultured on confocal dish at 2 × 10^4^ cells/dish. After treated as indicated, cells were loaded with 1.0 μM of calcein-acetomethoxy ester (Calcein AM) and 1.0 mM CoCl_2_ in Hanks’ balanced salt solution for 20 min at 37°C. Images were acquired using a confocal fluorescent microscope.

### Transmission Electron Microscopy

Transmission electron microscopy (TEM) was used to conduct morphological analyses and performed at the Army Medical University, according to their standard operating procedures. For mitochondrial detection, hearts were fixed in 2.5% glutaraldehyde (Sigma) in phosphate buffer overnight at 4°C and then 90–100 nm thick sections were mounted onto a 200-mesh copper grid. For MDV observation, the MDV suspension was deposited onto formvar carbon-coated TEM grids and then negatively stained with 2% uranyl acetate for 30 s. Samples were observed under a transmission electron microscope (TEM, HT7700, Hitachi, Tokyo, Japan) and imaged.

### Nanoparticle Tracking Analysis

For the measurement of the size of MDVs, 10 μL MDV sample was diluted in 1 mL of PBS and then analyzed using the nanoparticle tracking system (Malvern Panalytical, Malvern, United Kingdom).

### MDV Fluorescent Labeling and MitoTracker Staining

PKH26 is a membrane dye that intercalates the aliphatic portion of exposed lipid bilayers, and has been widely used to label extracellular vesicles (Cao et al., 2019). Given that MDVs were also membrane structures, we labeled MDVs using a PKH26 Red Fluorescent Cell Linker kit according to the manufacturer’s protocol. A total of 2 μL of PKH26 was mixed with 500 μL Diluent C. MDVs were resuspended in 500 μL Diluent C and then added to the above mixture. After 4 min of incubation at room temperature, 3% bovine serum albumin was added. The labeled MDVs were then centrifuged at 110,000 × *g* for 80 min at 4°C and re-suspended in PBS prior to uptake assays.

Mitochondrial morphology was evaluated using MitoTracker Green FM according to the manufacturer’s protocol. Briefly, H9C2 cells were incubated with DMEM supplemented with 50 nM MitoTracker Green FM in the dark for 30 min. After three washes with medium without FBS, the cells were used for subsequent experiments.

For the uptake assay, H9C2 cells were incubated with labeled MDVs for 2 h. Images were obtained using an inverted fluorescence microscope.

### Sucrose Density-Gradient Centrifugation

Cells were homogenized, and the supernatant was fractionated on a discontinuous sucrose gradient. Briefly, 1 mL of 60% sucrose was loaded at the bottom of an ultracentrifuge tube, followed by 1 mL of 50, 40, 30, 20, 10% sucrose, and 4 mL of supernatant. Gradients were centrifuged at 210,000 × *g* for 12 h. Fractions were then collected in SDS-PAGE sample buffer. All procedures were performed at 4°C.

### Statistical Analysis

Statistical analyses were performed using GraphPad Prism 5 software package (La Jolla, CA, United States). Data are presented as means ± SD of at least three independent experiments. Multiple-group statistical analyses were performed by one-way ANOVA followed by Bonferroni’s multiple comparison *post hoc* test. If data failed to follow a normal distribution, a Mann-Whitney Rank Sum test was performed. The significance level was defined as *P* < 0.05.

## Results

### MDV Production Is Negatively Correlated With Cardiomyocyte Apoptosis During Hypoxic Stress

**FIGURE 1:**
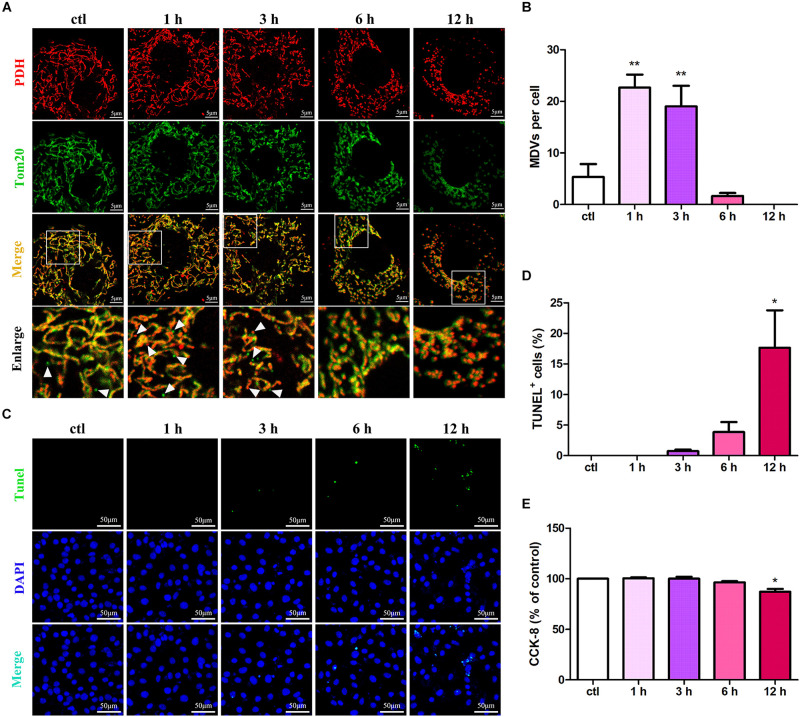
MDV production negatively correlates with cardiomyocyte apoptosis under hypoxic conditions. **(A,B)** H9C2 cells were treated with hypoxia for different times (0, 1, 3, 6, or 12 h) and immunolabeled for TOM20 (green) and PDH (red). MDV production increased in a time-dependent manner following exposure to hypoxia with a peak at 1 h and then decreased gradually; *P* values were estimated using one-way ANOVA with Bonferroni’s *post hoc* test; **P < 0.01 versus 0 h (ctl). **(C,D)** H9C2 cells were treated with hypoxia as indicated, and cell apoptosis was detected by TUNEL staining. The rate of apoptotic cells increased in a time-dependent manner with a significant increase in apoptosis at 12 h (*n* = 3); *P* values were estimated using one-way ANOVA with Bonferroni’s *post hoc* test; *P < 0.05 versus 0 h (ctl). **(E)** H9C2 cells were treated with hypoxia as indicated, and cell viability was evaluated using CCK-8. Cell viabilities decreased in a time-dependent manner with a significant decrease at 12 h (*n* = 3); *P* values were estimated using one-way ANOVA with Bonferroni’s *post hoc* test; *P < 0.05 versus 0 h (ctl).

To evaluate the roles of MDVs in H9C2 cardiomyocytes during hypoxic stress, we first evaluated changes in MDV production in hypoxia-treated H9C2 cells using immunofluorescence staining. Those vesicles that were 300 nm in size and PDH or TOM20 positive were classified as MDVs. As shown in Figures 1A,B, both PDH^+^ and TOM20^+^ MDV production increased in a time-dependent manner after exposure to hypoxic conditions with a peak at 1 h and then decreased gradually. After 12 h hypoxia treatment, almost no MDVs were shown in the cells. Next, we examined cell apoptosis in response to hypoxia using the TUNEL assay. Results revealed that the number of apoptotic cells significantly increased after 12 h of hypoxia (Figures 1C,D). Moreover, the cell viability of H9C2 under hypoxic conditions was tested using the CCK-8 assay and, as expected, cell viability results were consistent with the results from the cell apoptosis analyses (Figure 1E). These results suggest that MDV production is negatively correlated with cardiomyocyte apoptosis during hypoxic stress.

### Exogenous MDVs Mitigate Hypoxia-Induced H9C2 Cardiomyocyte Apoptosis

**FIGURE 2:**
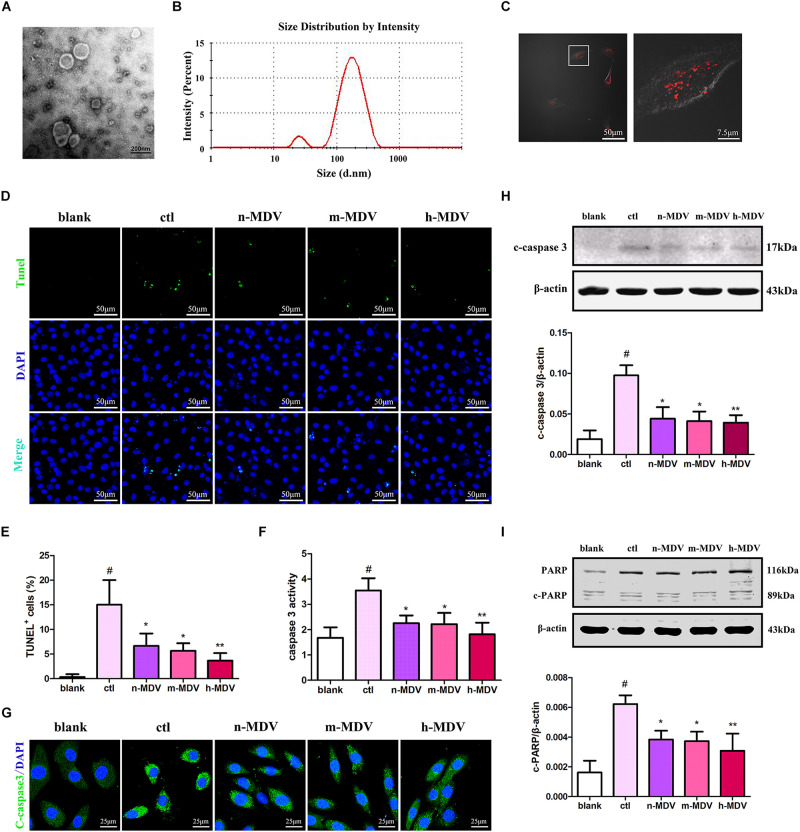
Exogenous MDVs mitigate hypoxia-induced H9C2 cardiomyocyte apoptosis. **(A)** Transmission electron microscopy images of the MDVs. **(B)** Results of particle size analysis showed that MDVs were predominantly ∼50–150 nm in size. **(C)** H9C2 cells were treated with PKH26 (red) labeled MDVs (12 μg/mL) for 2 h. Reconstituted hypoxic MDVs were created from mitochondria isolated from *ex vivo* isolated Langendorff-perfused rat hearts and grouped into normoxic- (n-MDVs), mild hypoxic- (m-MDVs), and heavy hypoxic MDVs (h-MDVs). H9C2 cells were treated as indicated for 12 h: blank (without hypoxia), ctl (hypoxia alone), n-MDV (hypoxia + n-MDVs), m-MDV (hypoxia + m-MDVs), and h-MDV (hypoxia + h-MDVs). **(D,E)** Hypoxia significantly increased the number of apoptotic cells, which was improved by the addition of the MDVs (*n* = 3); *P* values were estimated using one-way ANOVA with Bonferroni’s *post hoc* test; ^#^*P* < 0.05 versus blank, *P < 0.05 versus ctl, **P < 0.01 versus ctl. **(F)** Hypoxia treatment significantly increased caspase 3 activity, while all three types of MDVs reversed this trend (*n* = 3); *P* values were estimated by one-way ANOVA with Bonferroni’s *post hoc* test; ^#^*P* < 0.05 versus blank, *P < 0.05 versus ctl, **P < 0.01 versus ctl. **(G)** H9C2 cells were treated as indicated for 12 h and immunolabeled to identify cleaved-caspase 3 (c-caspase 3, green) and nuclear material (DAPI-blue). Hypoxia significantly upregulated the expression of c-caspase 3, which could be reversed by treatment with any of the three types of MDVs. **(H)** H9C2 cells were treated as indicated for 12 h and expression of cleaved-caspase 3 (c-caspase 3) was detected by Western blot. Hypoxia significantly upregulated the expression of c-caspase 3, which could be reversed by all three types of MDVs (*n* = 3); *P* values were estimated using one-way ANOVA with Bonferroni’s *post hoc* test; ^#^*P* < 0.05 versus blank, *P < 0.05 versus ctl, **P < 0.01 versus ctl. **(I)** Hypoxia significantly upregulated the expression of cleaved-PARP (c-PARP), while treatment with MDVs could partially reverse this trend (*n* = 3); *P* values were estimated by one-way ANOVA with Bonferroni’s *post hoc* test; ^#^*P* < 0.05 versus blank, *P < 0.05 versus ctl, **P < 0.01 versus ctl.

To determine the possible roles of MDVs in H9C2 cells during hypoxia, we reconstituted MDVs *in vitro*. The mitochondria isolated from heart tissues were intact and functional prior to their application in this experiment (Supplementary Figure 1). Results of the *in vitro* MDV reconstitution demonstrated that our *ex vivo* MDVs maintained membrane-bound vesicular profiles (Figure 2A) and had a size distribution ranging from 50 to 150 nm (Figure 2B). To imitate the intracellular role of MDVs, we first investigated whether exogenous MDVs could be taken up by H9C2 using PKH26-labeled (Red) MDVs. As shown in Figure 2C, after 2 h of treatment, PKH26-labeled MDVs [12 μg/mL, this concentration was referred to a previous studies (Burger et al., 2015; Jiang et al., 2018)] were dispersed throughout the cytoplasm of the H9C2 cells. In order to clarify whether exogenous MDVs could modulate hypoxia-induced H9C2 apoptosis and whether there were differences in the effects of MDVs produced by different hypoxic conditions on hypoxic cardiomyocyte apoptosis, we used perfused heart to fully mimic the mitochondrial environment in hypoxic tissues and observe the effects of exogenous MDV treatments. According to the O_2_ levels in the Tyrode solutions, MDVs were divided into normoxic MDVs (n-MDVs), mild hypoxic MDVs (m-MDVs), and heavy hypoxic MDVs (h-MDVs). As shown in Figures 2D,E, hypoxia significantly increased the rate of apoptotic cells, which was reduced following MDV treatment. There was a trend that suggests that h-MDVs exerted better protective effects during hypoxia. Given that the induction of apoptosis and activation of proapoptotic proteins result in caspase activation (cysteine proteases), we investigated whether hypoxia-induced cardiomyocyte apoptosis was caspase-dependent. As shown in Figures 2F–H, hypoxia increased caspase 3 activity and the expression of cleaved-caspase 3, which could be reversed by MDV treatment. In addition, we detected another commonly used apoptotic protein marker, cleaved-PARP. As shown in Figure 2I, following 12 h of hypoxia treatment, cleaved-PARP expression was significantly upregulated, and MDV treatment was able to moderately inhibit this upregulation. These results suggest that exogenous MDVs exert protective effects against hypoxia-induced cardiomyocyte apoptosis, which seems to correlated with the degree of hypoxia used to induce MDV production.

### Exogenous MDVs Protect Against Hypoxia-Induced H9C2 Cardiomyocyte Apoptosis via the Mitochondrial Pathway

**FIGURE 3:**
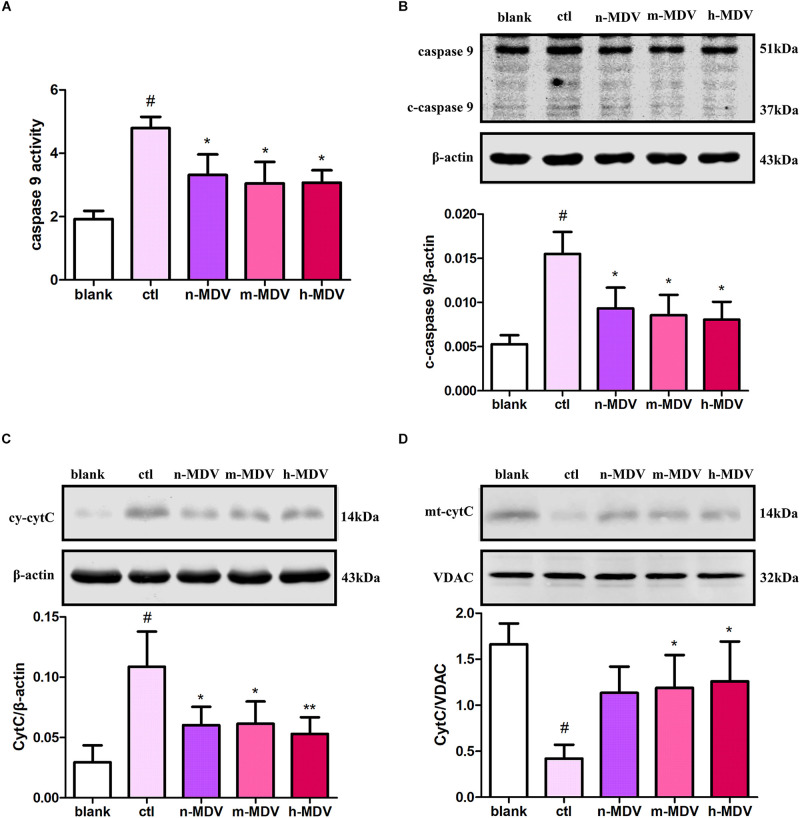
Exogenous MDVs protect against hypoxia-induced H9C2 cardiomyocyte apoptosis via the mitochondrial pathway. H9C2 cells were treated as follows for 12 h: blank (without hypoxia), ctl (hypoxia alone), n-MDV (hypoxia + n-MDVs), m-MDV (hypoxia + m-MDVs), and h-MDV (hypoxia + h-MDVs). **(A)** Hypoxia significantly increased caspase 9 activity, with all three types of MDVs reversing this trend (*n* = 3); ^#^*P* < 0.05 versus blank, *P < 0.05 versus ctl. **(B)** Hypoxia significantly upregulated the expression of cleaved-caspase 9 (c-caspase 9), and treatment with MDVs could reverse some of this increased expression (*n* = 3); *P* values were estimated using one-way ANOVA with Bonferroni’s *post hoc* test; ^#^*P* < 0.05 versus blank, *P < 0.05 versus ctl. **(C,D)** Hypoxia significantly upregulated the expression of cytoplasmic CytC and reduced the expression of mitochondrial CytC, both of which were reversed following treatment with MDVs (*n* = 3); *P* values were estimated using one-way ANOVA with Bonferroni’s *post hoc* test; ^#^*P* < 0.05 versus blank, *P < 0.05 versus ctl, **P < 0.01 versus ctl.

Because mitochondria play important roles in apoptotic cell death resulting from various stimuli, we investigated whether exogenous MDVs protect against hypoxic cardiomyocyte apoptosis via this pathway. Cytochrome c (CytC) release and induced caspase 9 activation are considered key events in mitochondria-dependent apoptosis, and both were detected in this study. These results demonstrate that hypoxia significantly increased caspase 9 activity, which could be reversed by all three types of MDVs (Figure 3A). As shown in Figure 3B, the expression of cleaved-caspase 9 was increased after hypoxia treatment, and all three MDVs showed moderate reductions in its expression, although none could completely reverse this phenotype. Moreover, hypoxia significantly upregulated the expression of cytoplasmic CytC and reduced mitochondrial CytC, both of which were reversed following the addition of the MDVs (Figures 3C,D). These results suggest that exogenous MDVs play a protective role in hypoxic cardiomyocyte apoptosis via the mitochondrial pathway.

**FIGURE 4:**
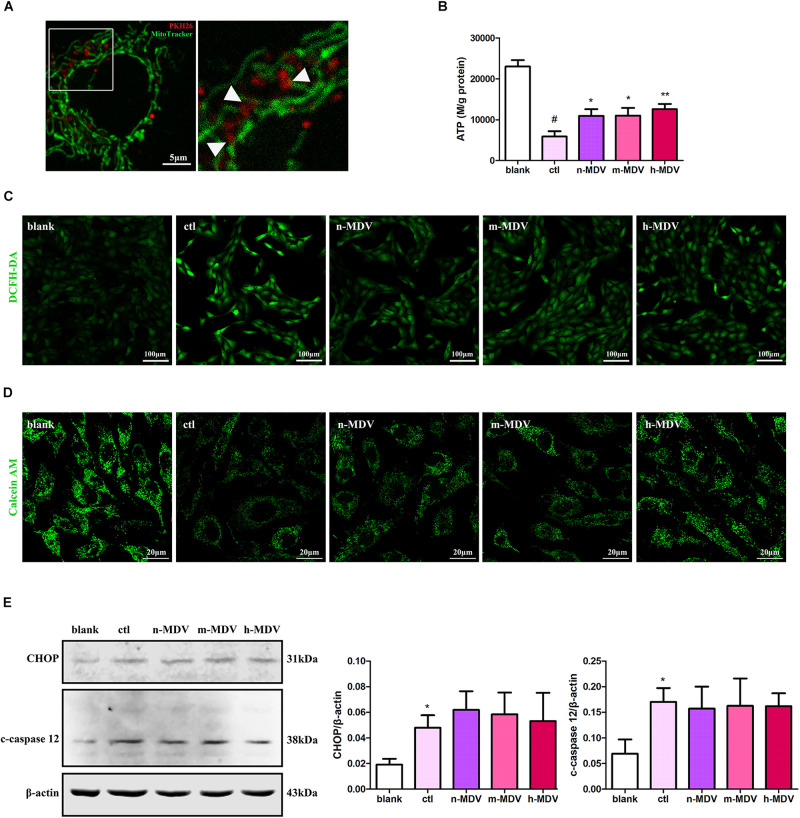
Exogenous MDVs exert direct protective effects on mitochondria. **(A)** Representative images of the colocalization of MDVs labeled with PKH26 (red) and mitochondria labeled with MitoTracker Green (green). **(B)** H9C2 cells were treated as indicated for 12 h. Hypoxia significantly reduced intracellular ATP levels, which could be reversed following treatment with any of the three types of MDVs (*n* = 3); *P* values were estimated by one-way ANOVA with Bonferroni’s *post hoc* test; ^#^*P* < 0.05 versus blank, *P < 0.05 versus ctl, **P < 0.01 versus ctl. **(C)** H9C2 cells were treated as indicated for 12 h and ROS generation was evaluated using DCFH-DA dye. Hypoxia significantly increased intracellular ROS levels, which could be reversed by any of the three types of MDVs. **(D)** H9C2 cells were treated as indicated for 3 h and mPTP opening was assessed by co-loading calcein AM and CoCl_2_. Opening of mPTP was significantly enhanced following hypoxia and was seen to significantly decrease following the addition of MDVs. **(E)** H9C2 cells were treated as indicated for 12 h. Hypoxia significantly upregulated the expression of CHOP and cleaved-caspase 12 (c-caspase 12), but no significant changes were observed following the addition of any of the three types of MDVs (*n* = 3); *P* values were estimated using one-way ANOVA with Bonferroni’s *post hoc* test; *P < 0.05 versus blank.

To further clarify the specific mechanisms used by these exogenous MDVs to protect against hypoxic cardiomyocyte apoptosis, we first detected the co-location of MDVs and mitochondria. As shown in Figure 4A, there were large numbers of MDVs co-localized with mitochondria. Next, we detected ROS production and intracellular ATP levels to clarify the effect of exogenous MDV treatment on mitochondrial function. As shown in Figures 4B,C, exogenous MDVs inhibited hypoxia-induced ROS production and upregulated intracellular ATP levels. The opening of mPTP is one of the mechanisms that contribute to mitochondrial apoptosis and ROS generation. The effect of exogenous MDV treatment on mPTP opening was analyzed by observing the changes in calcein fluorescence. Hypoxia significantly reduced the fluorescence intensity of mitochondrial calcein, indicating mPTP opening. All three types of MDVs reversed this effect and trend data suggests that h-MDVs exerted a better protective effect than the other MDV constructs (Figure 4D). In addition to mitochondria, endoplasmic reticulum (ER) also participates in apoptosis regulation, and there is close cross-talk between the mitochondria and ER. Therefore, the expression of CHOP and cleaved-caspase 12 was also evaluated to determine the extent of ER-mediated apoptosis. As shown in Figure 4E, the expression of CHOP and cleaved-caspase 12 was significantly upregulated after hypoxia, but no change was observed for these parameters following the addition of the exogenous MDVs. These results suggest that exogenous MDVs exert protective effects via the mitochondrial pathway rather than the ER pathway.

### Exogenous MDVs Protect Against Hypoxia-Induced H9C2 Cardiomyocyte Apoptosis by Conveying Bcl-2

**FIGURE 5:**
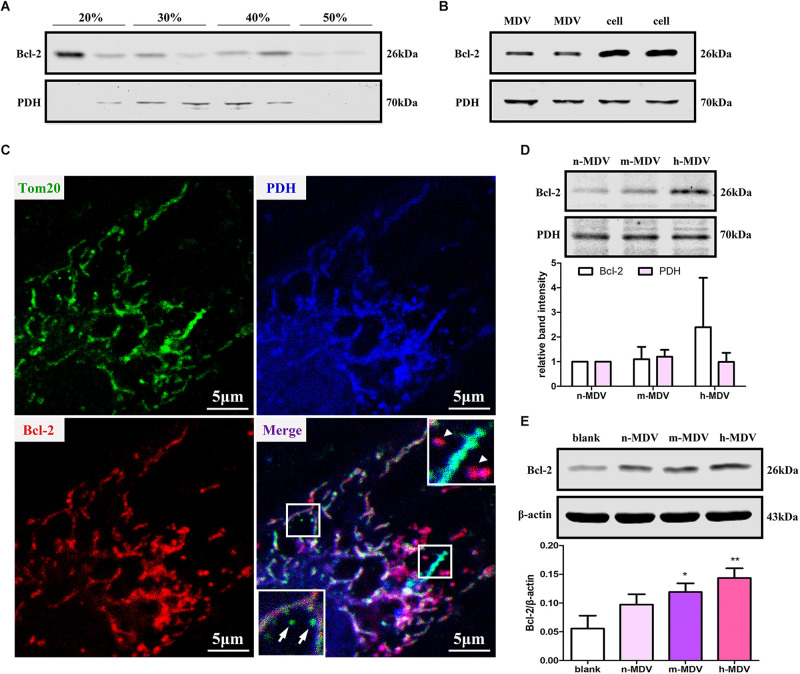
Exogenous MDVs protect against hypoxia-induced H9C2 cardiomyocyte apoptosis by conveying Bcl-2. **(A)** H9C2 cells were subjected to hypoxic conditions for 1 h and then homogenized for fractionation. Light fraction containing Bcl-2 appeared at the 20/30% sucrose interface and a heavier fraction containing Bcl-2 sedimented at the 40/50% interface. **(B)** Proteins were collected from MDVs for Western blot (whole cell protein served as a control). **(C)** H9C2 cells were treated with hypoxia for 1 h and immunolabeled for TOM20 (green), Bcl-2 (red), and PDH (blue). TOM20^+^ vesicles could be observed (white arrow); Bcl-2^+^/PDH^+^ vesicles (white triangle) rather than Bcl-2^+^/TOM20^+^ vesicles were observed. **(D)** Proteins were collected from different types of MDVs, and equal quantities of MDV proteins were used for Bcl-2 detection. **(E)** H9C2 cells were treated with the three types of MDVs and total proteins were collected, showing that cells treated with MDVs had more Bcl-2 than the control (*n* = 3); *P* values were estimated using one-way ANOVA with Bonferroni’s *post hoc* test; *P < 0.05 versus blank, **P < 0.01 versus blank.

Bcl-2 plays an important role in mitochondria-dependent apoptosis (Popgeorgiev et al., 2018) and Bcl-2 may escape from the mitochondria via vesicle transportation after treatment with mitochondrial uncoupler carbonyl cyanide m-chlorophenylhydrazone (CCCP) (Saita et al., 2013). We first determined whether Bcl-2 was enriched on membranous structures during hypoxia treatment. After 1 h hypoxia treatment, cells were homogenized, and the supernatant was fractionated on a discontinuous sucrose gradient. The results showed that a light fraction containing Bcl-2 appeared at the 20/30% sucrose interface and a heavier fraction containing Bcl-2 sedimented at the 40/50% interface, while established MDV cargo PDH was enriched at the 30/40% sucrose interface (Figure 5A). Second, we confirmed that Bcl-2 was present in MDVs. As expected, immunoblot analysis showed that Bcl-2 was present in MDVs, along with PDH (Figure 5B). Furthermore, confocal immunofluorescence imaging confirmed the presence of Bcl-2^+^/PDH^+^ vesicles, with relatively few Bcl-2^+^/TOM20^+^ vesicles after 1 h of hypoxia (Figure 5C). Next, we determined whether the levels of Bcl-2 were different among different MDVs produced by different hypoxia conditions. Equal quantities of MDV protein was resolved by SDS-PAGE. Results showed that there was a trend of increasing Bcl-2 concentration with increasing hypoxia, with the highest concentration of Bcl-2 in h-MDVs, while all three types of MDVs contained similar quantities of PDH (Figure 5D). In addition, cells were treated with different hypoxia-induced MDVs, and total proteins were collected. The results showed that cells treated with MDVs had more Bcl-2 than the control, and there was a trend of higher Bcl-2 concentrations in cells treated with h-MDVs (Figure 5E). These results suggest that exogenous MDVs protect against hypoxia-induced H9C2 cardiomyocyte apoptosis by conveying Bcl-2.

### Exogenous MDVs Show a Marginal Protective Effect on Myocardial Injury in Acute Ischemic Animals

**FIGURE 6:**
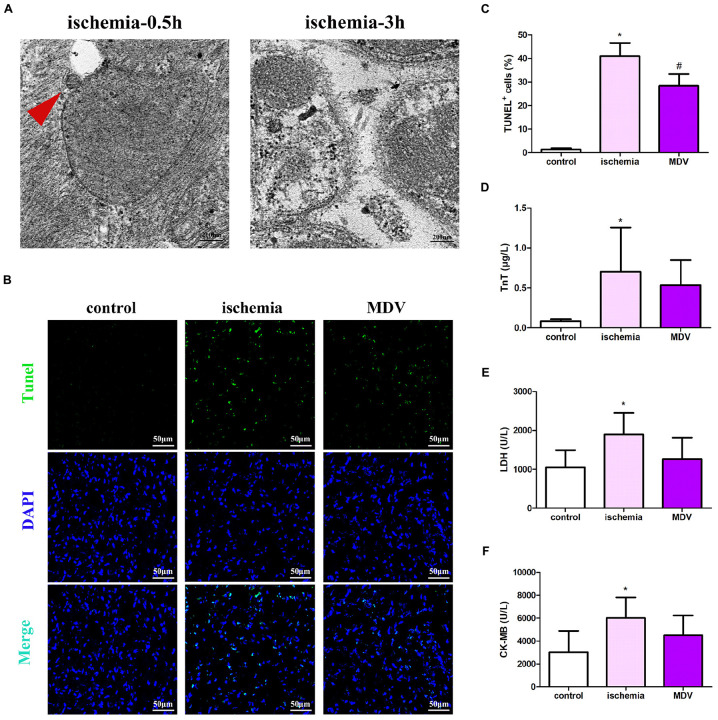
Effect of exogenous MDVs on myocardial injury in general ischemic animals. **(A)** Transmission electron microscopy images of the heart tissues from the general ischemic rats. **(B,C)** TUNEL staining of the cardiac tissues (*n* = 3); *P* values were estimated by one-way ANOVA with Bonferroni’s *post hoc* test; *P < 0.05 versus control, #*P* < 0.05 versus ischemia. **(D–F)** Statistical histogram of TnT, LDH, and CK-MB levels in each group (*n* = 6 per group); *P* values were estimated by one-way ANOVA with Bonferroni’s *post hoc* test; *P < 0.05 versus control.

To investigate the role of MDVs in myocardial injury in traumatic hemorrhagic shock animals, we first evaluated changes in MDVs produced by the heart tissues of hemorrhagic shock rats. As shown in Figure 6A, mitochondrial vesicle budding was frequently observed in hearts following 0.5 h of traumatic shock but not in animals subjected to 3 h of traumatic shock. Next, h-MDVs (600 μg/kg) were injected into these general ischemic rats, and the apoptosis levels of the myocardial cells and myocardial injury index were determined. As shown in Figures 6B,C, after general ischemic injury in rats, the number of apoptotic myocardial cells increased, while h-MDV treatment reduced the number of apoptotic myocardial cells when compared to the ischemia group. As shown in Figures 6D–F, TnT, LDH, and CK-MB level all increased in the shock groups. There were trends that suggest that h-MDVs may reverse this damage, although there was no significant statistical difference between these groups. These results suggest that exogenous MDV treatment may improve myocardial injury in general ischemic animals, although these findings need to be more carefully evaluated to make a definitive call.

## Discussion

**FIGURE 7:**
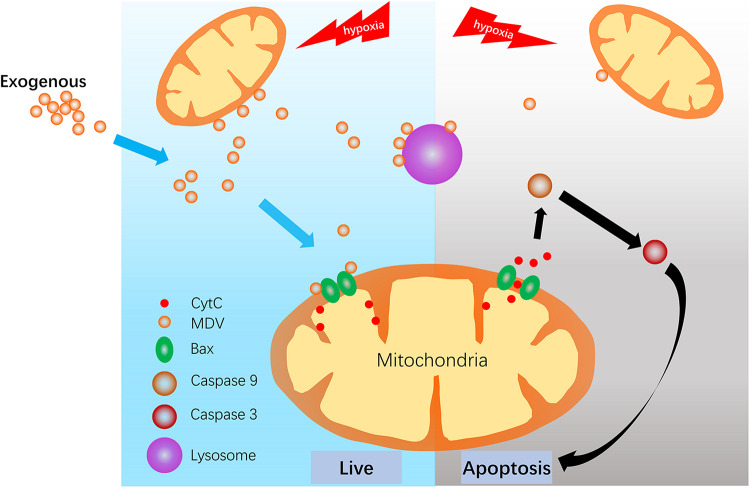
Schematic depiction of the potential protective effect of MDVs during hypoxic stress.

Cardiomyocyte apoptosis is the main form of hypoxic myocardial injury for various conditions including trauma-induced hemorrhagic shock. Understanding the specific underlying mechanisms of this response is important for improving the treatment of hypoxic myocardial injuries. This study demonstrated that MDV production was negatively correlated with cardiomyocyte apoptosis during hypoxia and exogenous MDVs could inhibit hypoxia-induced cardiomyocyte apoptosis and protect against hypoxia-induced cardiomyocyte apoptosis via the mitochondrial pathway by conveying Bcl-2. Moreover, it seems that exogenous MDVs from heavy hypoxia-induced myocardial tissues exert the best protective effect (Figure 7).

MDVs are membranous structures secreted by mitochondria and characterized by independent drp1-mediated cleavage mechanisms, which contain specific mitochondrial contents and are between 70–150 nm in size (Sugiura et al., 2014). It is well accepted that MDVs are of functional importance in mitochondrial homeostasis. In the heart, MDVs are produced even under healthy conditions and are rapidly upregulated in response to stress (Cadete et al., 2016). It is suggested that there is constant MDV production in cardiac tissues and that these vesicles act as a first line of defense against stress. However, the role of MDVs in myocardial ischemic or hypoxic injury is unclear. Our study showed that both PDH^+^ and TOM20^+^ MDV production increased in a time-dependent manner after hypoxia, with a peak at 1 h and then decreased gradually. Moreover, MDV production was negatively correlated with cardiomyocyte apoptosis following hypoxia stress. Similarly, mitochondrial vesicle budding was observed in hearts from rats exposed to general ischemic injury for 0.5 h but not in animals exposed to 3 h of injury. It is suggested that MDVs play an important role in cardiomyocyte apoptosis induced by hypoxia/ischemia. However, whether there is a causal relationship between MDV production and apoptosis needs to be evaluated.

To date, no MDV-specific factors essential for their formation have been identified, which limits the study of their role in cardiac tissues. Given the fact that MDVs and exosomes possess similar membranous structures and sizes, we referred to the studies of exosomes (Wan et al., 2019) to create the experimental design for our analysis of exogenous MDV treatment. As expected, exogenous MDVs could be taken up by cardiomyocytes. The addition of exogenous MDVs could mitigate hypoxia-induced cardiomyocyte apoptosis. An *in vivo* study also showed that there were minor improvements in the myocardial injury index after injection of MDVs, although there was no statistical difference between the groups. Moreover, our study demonstrated that MDVs derived from heavy hypoxia-treated mitochondria had a marginally better protective effect against hypoxia than other MDVs.

It should be noted that the highest MDV production was observed at 1 h and the lowest MDV production at 12 h after hypoxic treatment. This seems to contradict the best protective effect of MDVs which were derived from heavy hypoxia-treated mitochondria. In fact, the O_2_ concentration during the cellular experiment was 0.2%, which indicates heavy hypoxia. Thus, by combining the time-course data and the effect of exogenous MDVs, we speculate that production of MDVs exerts an endogenous protective effect during the early phases of hypoxia. After prolonged hypoxia, MDV production is decreased, which reduces their concentrations and thus their protective effect resulting in cell death. However, the mechanisms responsible for MDV formation in the early phases of hypoxia and MDV degradation following prolonged exposure to hypoxic conditions remain to be explored. Rab9 appears to be the prime candidate for the mediation of this effect, as it plays a critical role in MDV production (Matheoud et al., 2016) and myocardial ischemia (Saito et al., 2019).

There are two regulatory pathways controlling cellular apoptosis, an external and an internal pathway (Savitskaya and Onishchenko, 2015). The external pathway, also called the death receptor pathway, is initiated by the binding of death ligands to certain cell surface receptors. In contrast, the internal pathway, also known as the mitochondrial pathway, involves the release of mitochondrial intermembrane proteins (including cytochrome c and Smac/Diablo) into the cytosol, resulting in activation of caspases 9 and 3, which is followed by intranuclear lamina degradation and reduced DNA integrity. Our results showed that exogenous MDVs taken up by cardiomyocytes were distributed in the cytosol and a large number of them co-localized with mitochondria. Moreover, MDVs reduced hypoxia-upregulated caspase 9 activity, CytC release, ROS production, and upregulated intracellular ATP levels, suggesting that MDVs play a protective role in hypoxic cardiomyocyte apoptosis through the mitochondrial pathway and help to maintain mitochondrial function. In addition, it is worth noting that the ER also participates in apoptosis regulation and there is extensive cross-talk between them. However, evidence from our study did not support the role of MDVs in mitigating ER regulated apoptosis.

Initial research on MDVs demonstrated that MDVs transport mitochondrial outer membrane anchoring protein ligase (MAPL) to the peroxisome, although the significance of this remains unknown (Neuspiel et al., 2008). Another study demonstrated that MDVs act as the fourth mechanism of mitochondrial quality control, transporting specific proteins to the lysosomes for degradation (Soubannier et al., 2012a). More recently, it was reported that MDVs could transport mitochondrial autoantigens to the surface of the cell membrane to trigger the immune response (Matheoud et al., 2016); MDVs have also been shown to deliver Sod2 to bacteria-containing phagosomes in order to produce hydrogen peroxide and kill invading bacteria (Abuaita et al., 2018). In addition, MDVs have been closely linked to exosomes via the fact that MDVs may enter the late endosome/multivesicular body, which could route these to the cell surface leading to the release of exosomes (Raposo and Stoorvogel, 2013). Thus, it is reasonable that protein, lipid, and non-coding RNAs could all be contained in MDVs. Considering that Bcl-2 plays important roles in mitochondria-dependent apoptosis and that Bcl-2 may escape from the mitochondria via vesicle transportation after mitochondrial uncoupler CCCP treatment (Saita et al., 2013), we evaluated whether Bcl-2 was the functional content in the hypoxia-induced MDVs. Results showed that during hypoxia, Bcl-2 could be transported by vesicular carriers. MDVs derived from heavy hypoxia-treated mitochondria contained more Bcl-2 and showed better anti-apoptotic effects than the other two types of MDVs, suggesting that the anti-apoptotic effects of MDVs in our study may be positively correlated with Bcl-2 concentration. Therefore, we speculate that Bcl-2-containing MDVs may act as a means of communication between mitochondria. Facing hypoxic stimulation, relatively healthy mitochondria or mitochondria with abundant Bcl-2 may transfer Bcl-2 to less healthy mitochondria via these MDVs. In these less healthy mitochondria, Bax and Bak are activated and accumulated in the mitochondrial outer membrane; they then oligomerize and mediate mPTP opening, leading to the release of proapoptotic factors (Lovell et al., 2008). Bcl-2 may bind BAX-forming hetero- or homodimers, which further suppresses apoptosis (Siddiqui et al., 2015). However, the reason Bcl-2 containing MDVs target the mitochondria remains unclear. Previous studies have shown that MDVs may target lysosomes (Soubannier et al., 2012a), peroxisomes (Neuspiel et al., 2008) or phagosomes (Abuaita et al., 2018), amongst others and that this targeting depends on the cargo included in the MDVs and on the as-yet-undefined MDV transport and delivery network which may include Rab GTPases. Thus, further studies are needed to clarify the specific mechanisms of Bcl-2 containing MDV transport and delivery.

There are some limitations to this study. First, MDV-specific factors essential for their formation have not been identified, which limits our understanding of their role *in vivo* during hypoxia. Second, we did not modulate Bcl-2 concentrations in these MDVs, which limits the number of conclusions that we can draw regarding its contribution to the protective effects of hypoxia-induced MDVs. Third, although we observed significant protective effects when hypoxic cells were treated with MDVs, no clear protective effects were observed in the animal experiments. One possible explanation may be the significant differences in the extracellular environment between the *in vitro* and *in vivo* models, suggesting that the metabolism of these MDVs is more complicated in animals than in cultured cells. Another possible explanation may be the cellular model used for experiments. It is reported that differentiated H9C2 cells may be a better model for cultured primary cardiomyocytes to improve translation and relevance.

## Conclusion

This study provides evidence that MDVs can protect heart tissues against hypoxic myocardial injury by inhibiting mitochondrial apoptosis via the partial conveyance of Bcl-2. Our study used a novel approach to extend our understanding of MDVs and highlights their function as an endogenous protective mechanism during hypoxic injury. Strategies that stimulate cardiomyocytes to produce cargo-specific MDVs, including Bcl-2 containing MDVs, could theoretically be helpful in the treatment of ischemic/hypoxic myocardial injuries.

## Data Availability Statement

All datasets generated for this study are included in the article/Supplementary Material.

## Ethics Statement

The animal study was reviewed and approved by the Laboratory Animal Welfare and Ethics Committee of the Third Military Medical University.

## Author Contributions

LL, TL, and BL conceived and designed the study, and analyzed the data. All authors performed the experimental procedures. BL and HZ drafted the manuscript. LL and TL revised the manuscript. LL acquired the financial support.

## Conflict of Interest

The authors declare that the research was conducted in the absence of any commercial or financial relationships that could be construed as a potential conflict of interest.

## Abbreviations

CK-MB: creatine phosphokinase-MB
CytC: cytochrome c
DMEM: Dulbecco’s modified Eagle’s medium
ER: endoplasmic reticulum
FBS: fetal bovine serum
LDH: lactate dehydrogenase
MDVs: mitochondrial-derived vesicles
mPTP: mitochondrial permeability transition pore
TnT: troponin T.
