# Corrigendum: Mitochondrial-derived vesicles protect cardiomyocytes against hypoxic damage

**DOI:** 10.3389/fcell.2025.1546582

**Published:** 2025-04-07

**Authors:** Binghu Li, Hongliang Zhao, Yue Wu, Yu Zhu, Jie Zhang, Guangming Yang, Qingguang Yan, Junxia Li, Tao Li, Liangming Liu

**Affiliations:** State Key Laboratory of Trauma, Burns and Combined Injury, Department 2, Research Institute of Surgery, Daping Hospital, Army Medical University, Chongqing, China

**Keywords:** mitochondrial-derived vesicles, myocardial ischemia, mitochondrial, hypoxia, apoptosis

In the published article, there was an error in Figure 6 as published. There was an error in pasting the figure of DAPI of the ischemia group in Figure 6B. The corrected Figure 6 and its caption “Effect of exogenous MDVs on myocardial injury in general ischemic animals. (A) Transmission electron microscopy images of the heart tissues from the general ischemic rats. (B and C) TUNEL staining of the cardiac tissues (n = 3); P values were estimated by one-way ANOVA with Bonferroni’s post-hoc test; * P < 0.05 versus control, # P < 0.05 versus ischemia. (D–F) Statistical histogram of TnT, LDH, and CK-MB levels in each group (n = 6 per group); P values were estimated by one-way ANOVA with Bonferroni’s post-hoc test; * P < 0.05 versus control.” appear below.

**FIGURE 6:**
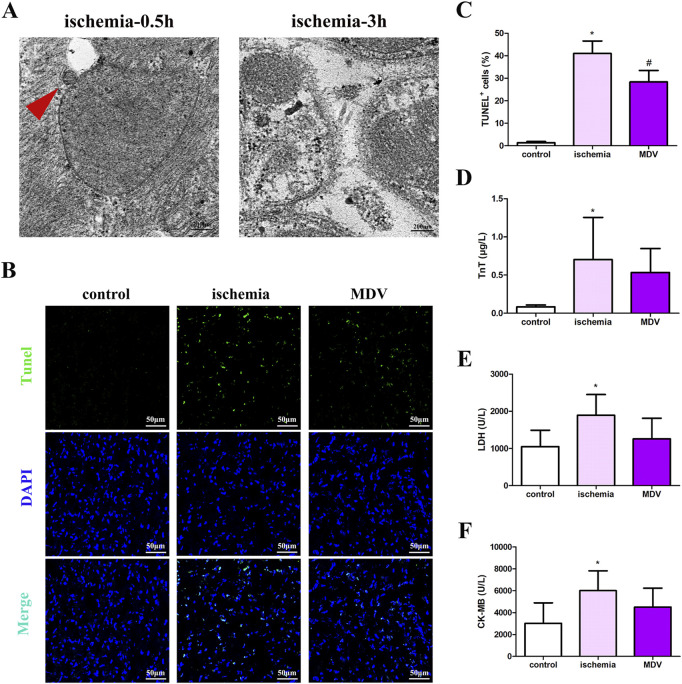
Effect of exogenous MDVs on myocardial injury in general ischemic animals. **(A)** Transmission electron microscopy images of the heart tissues from the general ischemic rats. **(B, C)** TUNEL staining of the cardiac tissues (n = 3); P values were estimated by one-way ANOVA with Bonferroni’s post-hoc test; * P < 0.05 versus control, # P < 0.05 versus ischemia. **(D–F)** Statistical histogram of TnT, LDH, and CK-MB levels in each group (n = 6 per group); P values were estimated by one-way ANOVA with Bonferroni’s post-hoc test; * P < 0.05 versus control.

The authors apologize for this error and state that this does not change the scientific conclusions of the article in any way. The original article has been updated.

