# Supplementary material for: Mitochondrial-Derived Vesicles Protect Cardiomyocytes Against Hypoxic Damage
Source: Front Cell Dev Biol. 2020 Apr 17;8:214. doi: 10.3389/fcell.2020.00214 (PMC7212461; doi:10.3389/fcell.2020.00214)
Supplement: Supplementary file 1 [file Image_1.pdf]

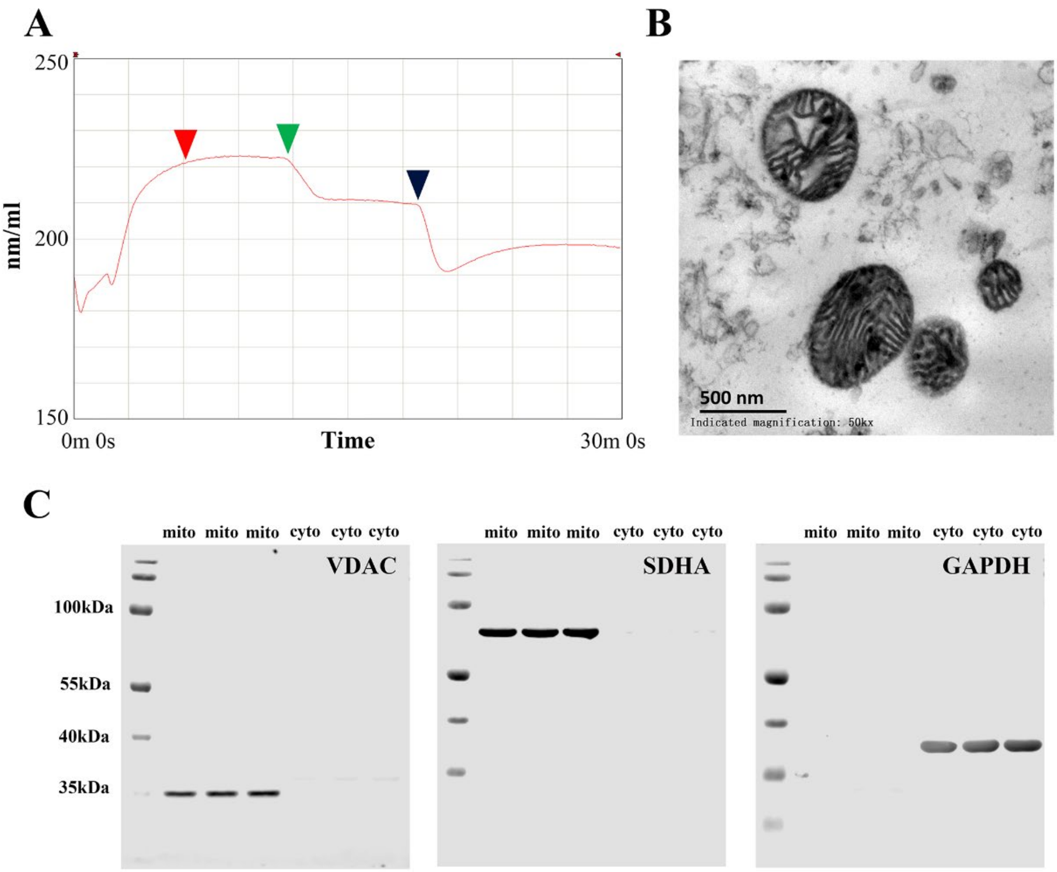


**Supplementary Figure 1 Identification and function detection of isolated mitochondria.** (**A**) Representative graph of isolated mitochondria oxygen consumptions showed that the isolated mitochondria were functional. Red triangle indicates the addition of mitochondria; green triangle indicates the addition of the mixture of malate sodium (0.5M) and L-glutamate sodium (0.5M); blue triangle indicates the addition of ADP (0.5M). (**B**) Transmission electron microscopy images of isolated mitochondria. (**C**) Western blot analysis of well-established mitochondrial makers, VDAC and SDHA. mito indicates mitochondrial protein; cyto indicates cytoplasmic protein.
